# Comparative proteomic analysis of differentially expressed proteins in the early milky stage of rice grains during high temperature stress

**DOI:** 10.1093/jxb/ert435

**Published:** 2013-12-27

**Authors:** Jiang-Lin Liao, Hui-Wen Zhou, Hong-Yu Zhang, Ping-An Zhong, Ying-Jin Huang

**Affiliations:** ^1^Key Laboratory of Crop Physiology, Ecology and Genetic Breeding (Jiangxi Agricultural University), Ministry of Education, Jiangxi Province 330045, China; ^2^Key Laboratory of Agriculture responding to Climate Change (Jiangxi Agricultural University), Nanchang City, Jiangxi Province 330045, China

**Keywords:** Early milky stage, high temperature, proteomics, rice, rice grain, two-dimensional electrophoresis (2-DE).

## Abstract

Rice yield and quality are adversely affected by high temperatures, and these effects are more pronounced at the ‘milky stage’ of the rice grain ripening phase. Identifying the functional proteins involved in the response of rice to high temperature stress may provide the basis for improving heat tolerance in rice. In the present study, a comparative proteomic analysis of paired, genetically similar heat-tolerant and heat-sensitive rice lines was conducted. Two-dimensional electrophoresis (2-DE) revealed a total of 27 differentially expressed proteins in rice grains, predominantly from the heat-tolerant lines. The protein profiles clearly indicated variations in protein expression between the heat-tolerant and heat-sensitive rice lines. Matrix-assisted laser desorption/ionization time-of-flight/time-of-flight mass spectrometry (MALDI-TOF/TOF MS) analysis revealed that 25 of the 27 differentially displayed proteins were homologous to known functional proteins. These homologous proteins were involved in biosynthesis, energy metabolism, oxidation, heat shock metabolism, and the regulation of transcription. Seventeen of the 25 genes encoding the differentially displayed proteins were mapped to rice chromosomes according to the co-segregating conditions between the simple sequence repeat (SSR) markers and the target genes in recombinant inbred lines (RILs). The proteins identified in the present study provide a basis to elucidate further the molecular mechanisms underlying the adaptation of rice to high temperature stress.

## Introduction

Climate change and abiotic stress affect agriculture and crop production adversely. Among the various climatic factors affecting agriculture, temperature is one of the most important because higher temperatures adversely affect plant growth and yield (Jagadish *et al.*, 2010; Chauhan *et al.*, 2011; Zou *et al.*, 2011; Madan *et al.*, 2012). In the 21st century, average surface temperatures of the Earth are likely to increase by 2–4.5 °C, with an increase in the magnitude and frequency of extreme temperature events (Peng *et al.*, 2004; Stevenson *et al.*, 2005). Therefore, it is imperative that crops are able to adjust to higher temperatures in the near future to be productive and survive.

Rice is one of the major cereal crops consumed by humans. Because it originated in tropical and subtropical areas, it has some ability to endure high temperatures. However, the growth of normal rice can be impaired in temperatures above a certain threshold (Welch *et al.*, 2010). The yield and quality of the japonica cultivars of rice are influenced by temperatures above 26 °C during the grain-filling stage (Peng *et al.*, 2004). High temperature stress in rice during the grain-filling stage results in an increased grain-filling rate, decreased grain weight, low amylose content, poor milling quality, and an increased degree of chalkiness (He *et al.*, 1990; Jiang *et al.*, 2003; Lin *et al.*, 2010). The physiological effects of higher temperatures on rice during the ripening period are generally well defined (Jiang *et al.*, 2003; Tang *et al.*, 2008). However, the molecular mechanisms triggering these effects are poorly understood (Yamakawa *et al.*, 2007; Yamakawa and Hakata, 2010).

The rice grain ripening period can be differentiated into two periods according to the ripening of the embryo and endosperm. During the ﬁrst half of the ripening period, rice embryos and endosperm undergo cell division and differentiation until the embryo reaches maturity, whereas the accumulation and transformation of starches and proteins take place in the second half of the ripening period (Hong *et al.*, 1995). Previous studies reported that high temperatures during the ﬁrst half of the ripening period mainly affect yield due to a decrease in grain plumpness. In contrast, high temperatures in the latter half of the ripening period predominantly affect quality due to impaired deposition and the transformation of storage materials such as starch content, starch components, and protein accumulation (Jiang *et al.*, 2003; Hakata *et al.*, 2012; Zhong *et al.*, 2012). This suggests that, since the process of rice kernel formation varies in the two stages of the ripening period, the molecular mechanisms underlying high temperature stress responses would also differ.

The identiﬁcation of the genes and proteins that are responsive to abiotic stresses is an essential step towards understanding the molecular mechanisms underlying the stress response (Khan and Komatsu, 2004; Liu and Xue, 2006). Several novel genes and proteins that respond to high temperature stress have been identified (Yamakawa *et al.*, 2007; Gammulla *et al.*, 2010; Yamakawa and Hakata, 2010; Liao *et al.*, 2012; Mittal *et al.*, 2012), which can assist in understanding the molecular mechanisms of this important crop.

Proteomics tools based on two-dimensional electrophoresis (2-DE) and mass spectrometry (MS) analyses provide an effective approach to investigate the molecular response of plants to stress. A previous study reported that >70 proteins in rice grains were differentially expressed when plants were exposed to high temperatures of 30–35 °C during the full-length grain-filling stage (Lin *et al.*, 2005). Other proteomic analyses of rice under high temperature stress conditions were also recently reported (Han *et al.*, 2009; Jagadish *et al.*, 2010; Lin *et al.*, 2010; Gammulla *et al.*, 2011). Advances in the proteome characteristics and the identiﬁcation of heat-responsive proteins in rice will help in understanding the regulatory network of this important crop. Although the ﬁrst half of the ripening period of rice is very sensitive to high temperature stress (Tang *et al.*, 2008; Chaki *et al.*, 2011; Li *et al.*, 2011), few functional proteins involved in the response to high temperature stress during the ﬁrst half of the ripening period have been identified.

To study the molecular mechanisms responsible for high temperature stress during the ﬁrst half of the ripening period, recombinant inbred lines (RILs), originating from Xieqingzao B/N22//Xieqingzao B, were bred (Liao *et al.*, 2011). Fifty-four candidate genes involved in the response of rice to high temperature stress during the ﬁrst half of the ripening period were identified using a heat-tolerant line (XN0437T) and its heat-sensitive counterpart (XN0437S) as the plant materials, which originated from the RILs described above (Liao *et al.*, 2011).

However, the molecular mechanisms of the response of plants to abiotic stress are highly complex. In addition, the regulation of gene expression occurs not only at the transcript level, but also at the protein level. The present study was conducted to determine the molecular mechanisms underlying the adaptation of rice to high temperature conditions, particularly during the first half of the ripening period. Therefore, XN0437T and XN0437S were used as the plant materials to identify the differentially displayed proteins in rice adapting to high temperature stress during the ﬁrst half of the ripening period. Genes encoding the differentially displayed proteins were further monitored on rice chromosomes using 887 simple sequemce repeat (SSR) markers and RIL populations.

## Materials and methods

### Plant materials

Two rice lines, heat-tolerant XN0437T and heat-sensitive XN0437S, were used as the plant materials in this study. They were inbred in a previous project, and showed significant differences in grain weight when exposed to high temperature during the ﬁrst half of the ripening period. These lines had a polymorphism rate of only 1.80% in the genome when the 887 SSR markers were used to detect the genetic polymorphism (Liao *et al.*, 2011).

### Growth conditions and treatments

The tub-planting method was used to culture the rice, as described previously (Liao *et al.*, 2012). Rice ears were labelled on the same heading date to ensure that only uniformly developed samples were used for analysis and to reduce the influence of artificial operation on the rice flowering process. On the 10th day after heading, plants with the same label were transferred to chambers and maintained at a temperature of 38.0±0.5 °C or 25.0±0.5 °C (treatment versus control). Rice seeds from the same region (middle to bottom part) of labelled ears were harvested at three different time points (1, 3, and 5 d) after the initiation of high temperature treatment, packed in aluminium foil, and flash-frozen in liquid nitrogen. Three biological replicates of each sample were taken for the high temperature treatment.

### Caryopsis weight and net photosynthetic rate

Caryopsis weight and the net photosynthetic rate of flag leaves were measured as described previously (Liao *et al.*, 2011, 2012). On the second day after treatment, variations in the net photosynthetic rate of rice flag leaves were measured using a portable infrared gas analyser system, LI-6400 (LI-COR., UK). Five leaves per sample were measured.

### Protein isolation, 2-D gel electrophoresis, SDS–PAGE, and gel staining

Total proteins were extracted from 3g of each caryopsis sample. Protein extraction, 2-D gel electrophoresis, SDS–PAGE, and gel staining were carried out as described previously (Liao and Huang, 2011). The isoelectric focusing (IEF) was carried out using 24cm IPG strips with a pH range of 5–8 NL (Bio-Rad, Hercules, CA, USA). Two-dimensional gels for image analysis were silver stained, and the differentially displayed proteins for MS were stained using Coomassie brilliant blue (CBB). Each gel was loaded with 190 μg of protein for silver staining, and with 1.2mg for CBB staining.

### Image analysis

Image analysis of the silver-stained 2-DE gels was carried out using Image Master 2D platinum software version 5.0 (Amersham Pharmacia Biotech, Uppsala, Sweden). Each gel was analysed for spot detection, background subtraction, and protein spot OD intensity quantification. The gel image showing the highest number of spots and the best protein pattern was selected as the reference gel. All spot comparisons and analyses were coordinated using the reference gel, and its spots were then matched across all gels. The value of each spot was normalized as a percentage of the total volume in all of the spots present in the gel to correct the variability due to the quantitative variations in the intensity of protein spots. The heat-responsive proteins were detected based on the criteria that (i) the differential spots were reproduced on three biological replicates; (ii) spot volume variations changed at least 2-fold at at least one time point between the treatment and control; and (iii) spot volume variations changed (*P* < 0.05) at at least at one time point between the heat-tolerant and heat-sensitive lines. The experimental molecular weight and isoelectric point (pI) of a protein were estimated using its position on the 2-D gel.

### MALDI-TOF/TOF MS analysis and database searching

The identified protein spots were excised from the CBB-stained gels and digested using trpysin. Digested proteins were further analysed using a matrix-assisted laser desorption/ionization time-of-flight/time-of-flight mass spectrometry (MALDI-TOF/TOF MS) 4700 proteomics analyser. The identities of the proteins were determined using MASCOT (Matrix Science, London, UK) software with the following optimized parameters: peptide mass tolerance of 100 ppm, a maximum of one missed tryptic cleavage, and allowing for iodoacetamide modiﬁcations including the oxidation of methionine. The peptide search was limited first to the taxonomy of rice. If no positive results were obtained, the search was performed again using green plants as the taxonomy. If peptides were matched to multiple members of a protein family, or if a protein appeared under different names and accession numbers, the entry with the highest score was selected. Identified proteins were grouped into individual categories according to their documented functions in MASCOT databases.

### Real-time qRT–PCR and data analysis

To investigate the corresponding mRNA expression patterns of the proteins differentially expressed during high temperature stress, eight genes were selected and their expression patterns were monitored further. Total RNA was isolated from 0.5g of caryopsis using the Column Plant RNAout2.0 Kit (Invitrogen, Carlsbad, CA,USA) following the manufacturer’s instructions. Double-stranded cDNA synthesis and the cDNA concentrations of all samples were normalized against the expression of the ACT1 gene (GenBank ID: AK100267) (Li *et al.*, 2010). The quantitative (qPCR) primers of the analysed genes were designed using the online primer software ‘primer-BLAST’ in NCBI according to the expressed sequence tag (EST) sequences of the genes encoding the identified proteins. Fragments after reverse transcription–PCR (RT–PCR) were then sequenced by Invitrogen at their China branch to confirm the identity of the products. Real-time qPCR and data analysis were then carried out as described previously (Liao *et al.*, 2012).

### Mapping identified genes to the rice genome

The RILs inbred in a previous study (Liao *et al.*, 2011) were used as the plant mapping populations to map the corresponding genes encoding differentially displayed proteins in the rice genome. A total of 887 SSR markers, uniformly distributed in various rice chromosomes, used to screen the genomic polymorphism in a previous study (Liao *et al.*, 2011) were used as mapping markers. If the PCR-amplified fragment of an identified gene co-segregated with one or more RIL markers, the identified genes were considered to be in the same linkage group as the markers, and therefore the identified gene could be mapped to the rice genome according to the locus of the markers. PCR primer pairs were designed using the online ‘Primer-BLAST’ software in NCBI according to the corresponding mRNA sequences of the differentially expressed proteins. The expected sizes of the PCR-amplified fragment sequences could be larger than the sizes of the target regions in the mRNA sequences, since an amplified region may contain intron(s). The PCR-amplified fragments were cloned into the pGEM-T Easy^®^ vector (Promega, Madison, WI, USA) and transformed into DH5α-competent cells (Invitrogen, USA) following the manufacturer’s instructions. The positive clones verified by PCR were sequenced at the China branch of Invitrogen to confirm the identity of the products.

### Statistical analyses

For all statistical analyses, at least three biological replicates were used for each treatment and control. The physiological results, the normalized values of protein spots, and the mRNA expression data from real-time RT–PCR were exported to the statistical software Data Processing System (DPS) version 14.10 (Zhejiang University, China), and a two-tailed Student’s *t*-test with a significance level of 95% was used.

## Results

### Effects of high temperatures on grain weight and net photosynthetic rate

It was reported that photosynthetic capacity and plant growth are strongly influenced by temperature (Nagai and Makino, 2009). In the present study, not only the chalkiness of rice (Supplementary Fig. S1 available at *JXB* online), but also the grain weight and net photosynthetic rate were decreased in both heat-tolerant (XN0437T) and heat-sensitive (XN0437S) rice lines after continued exposure to high temperatures (Supplementary Fig. S2). Compared with the heat-tolerant rice line, the net photosynthetic rate of the heat-sensitive rice line declined significantly (*P* < 0.05) after treatment with high temperature (38 °C) for 1 d. Several photosynthetic enzymes, such as Rubisco, undergo degradation in response to higher temperatures (>35 °C), which affects the photosynthetic rate (Crafts-Brandner and Salvucci, 2000, 2002). However, these enzymes may adapt to higher temperatures in heat-tolerant rice lines, accounting for their differential expression in the heat-tolerant and heat-sensitive rice lines.

### Protein profiling and analysis of protein changes

**Fig. 1.**
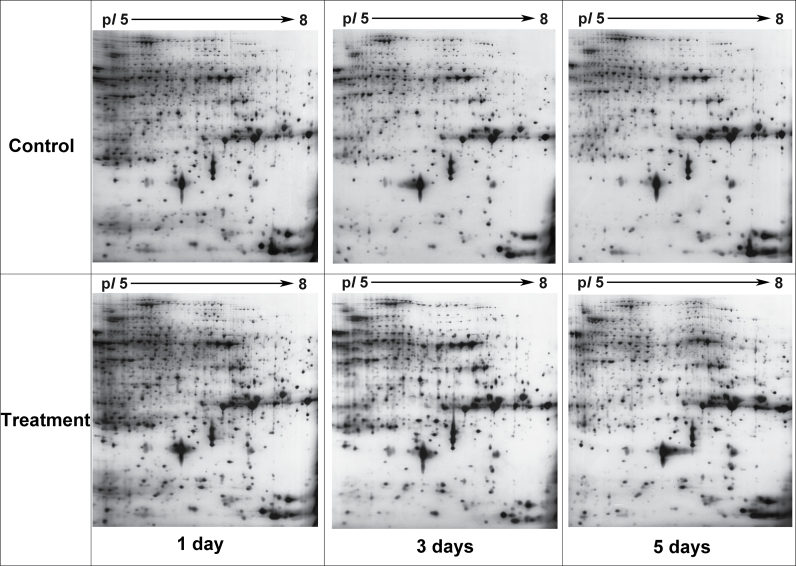
Six representative 2-DE profiles for the treatment and control samples of the heat-tolerant rice line exposed to high temperature for 1, 3, and 5 d. High temperature treatment for 1 d (A), 3 d (B), and 5 d (C).

Two-dimensional electrophoresis profiles showed that proteins isolated from young rice caryopsis were distributed throughout the gel, and >1100 reproducible protein spots were detected by Image Master 5.0 software after silver staining. Six representative 2-DE profiles for the treatment and parallel samples of the heat-tolerant rice line under high temperatures for 1, 3, and 5 d are shown in Fig. 1.

**Fig. 2.**
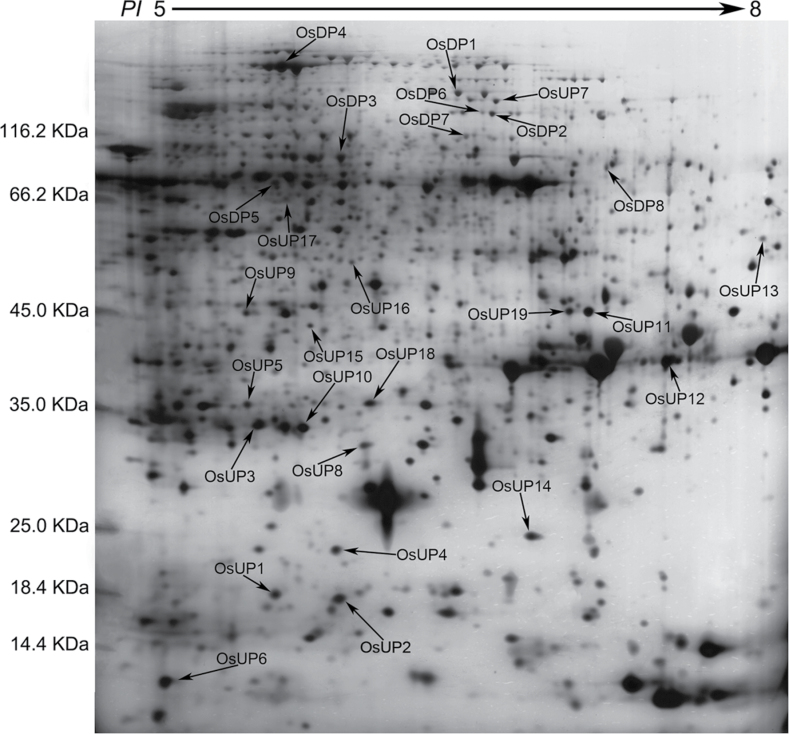
Location of differentially expressed proteins in the 2-DE profile. OsUP and OsDP indicate up- and down-regulated proteins, respectively. Molecular weights of the proteins are shown on the left of the 2-DE profile.

**Fig. 3.**
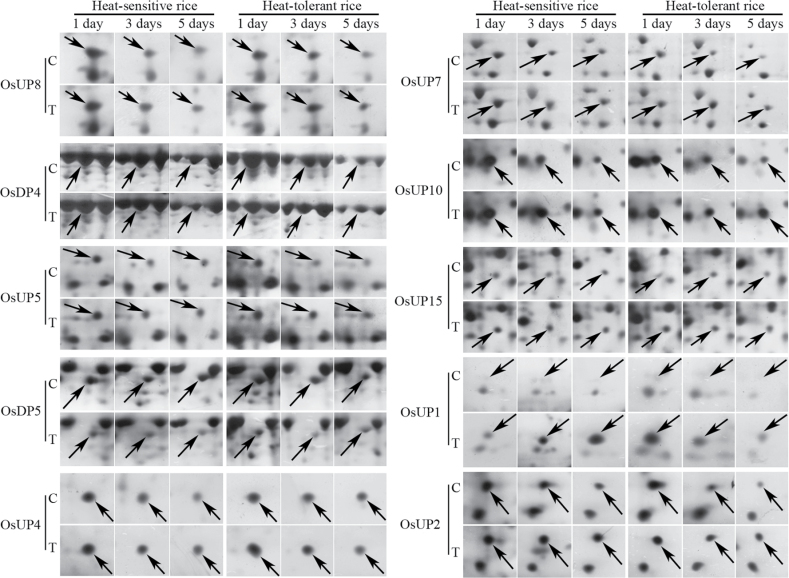
Ten representative proteins from the differentially expressed pattern from treated and control heat-tolerant and heat-sensitive rice lines under high temperature for 1, 3, and 5 d.

Based on the criteria used here for protein spot detection, 27 differentially expressed proteins were detected and their locations in 2-DE profiles are shown in Fig. 2. Among the 27 detected proteins, 19 proteins were up- and eight were down-regulated. In addition, the regulation range was significantly different (*P* < 0.05) between heat-sensitive and heat-tolerant rice lines at at least at one time point after high temperature stress. The differentially displayed proteins (with spot volume variations between treated and control samples from heat-tolerant and heat-sensitive rice lines) that could be distinguished visually are shown in Fig. 3. There were some differences between the experimental and theoretical molecular weight and pI of the identified proteins, which may be due to post-translational modification of the proteins and the use of non-linear pH gradients that can reduce the accuracy of pI estimation.

### Protein identification

During MALDI-TOF/TOF MS analysis, two spots (7.4%) out of the 27 detected proteins could not be identified conclusively, which could be attributed to low protein spot volume and/or low quality spectra. Several of the identified proteins showed discrepancies between their experimental and expected molecular weight, consistent with previous reports (Bonhomme *et al.*, 2009; Bedon *et al.*, 2012). This may be due to *in vivo* or *in vitro* protein degradation when the experimental molecular weight is lower, and post-translational modifications such as glycosylation when higher than expected experimental molecular weights were observed (Plomion *et al.*, 2006).

### Functional classification and expression profile analysis

All 25 proteins identified by MS showed homology with proteins of known function (Table 1). Based on the functions of homologous proteins, the 25 proteins were classified into five groups: 10 (40%) were likely to be involved in biosynthesis, four (16%) in energy metabolism, seven (28%) in oxidation, three (12%) in heat shock, and one (4%) in transcript regulation. Of the 25 proteins, 18 (72%) were up- and seven (28%) were down-regulated at one or more time points in heat-tolerant and/or heat-sensitive rice lines under high temperature stress.

**Table 1.** Identification, functional categorization, and quantitative analysis of the differentially displayed proteins

Spot Exp. name^*a*^	Protein score	Sequence coverage (%)	Peptide matches	Exp./Theo.		Protein hits	Protein identification	Changes in spot intensity^*b*^
				Mol. wt (kDa)	pI			
Biosynthesis								
OsUP8	290	22	10	31.7/56.9	6.21/8.93	gi|218165	Pre-pro-glutelin	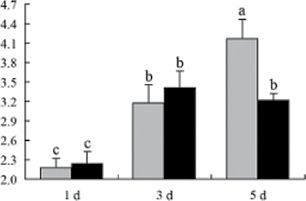
OsUP12	610	26	11	40.3/56.8	7.01/9.17	gi|169791	Glutelin	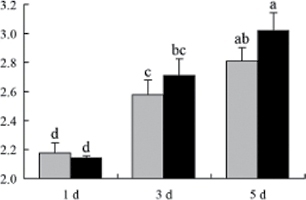
OsUP13	138	16	6	53.6/54.3	7.79/9.30	gi|218190412	Glutelin	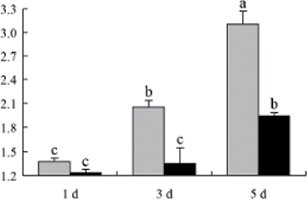
OsUP14	283	31	9	23.9/57.4	6.71/8.96	gi|115445979	Glutelin	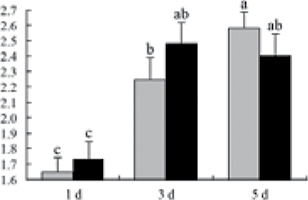
OsUP16	151	14	7	50.4/38.8	5.96/5.73	gi|22748337	Putative gln1_oryza glutamine synthetase isozyme	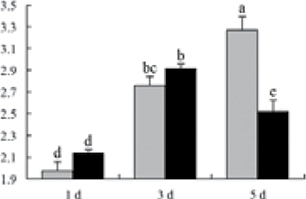
OsUP18	484	27	10	36.2/50.7	6.26/8.74	gi|213876598	Gt3, partial	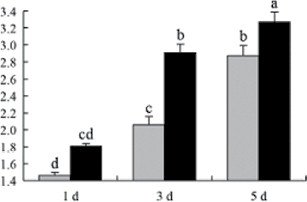
OsDP2	394	9	9	119.3/114.1	6.52/6.35	gi|218194315	Hypothetical protein OsI_14800 (pullulanase)	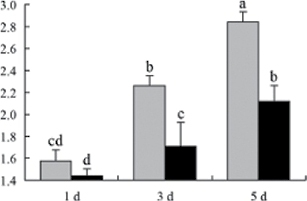
OsDP4	282	23	13	146.7/103.6	5.47/5.98	gi|115463815	Orthophosphate dikinase	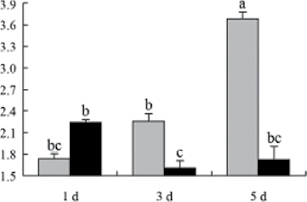
OsDP6	193	5	7	121.1/69.5	6.49/6.61	gi|115470493	Starch debranching enzyme	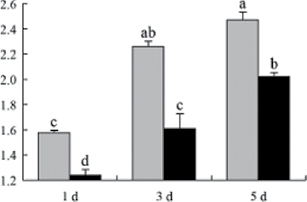
OsDP8	178	28	11	93.4/67.0	6.92/8.16	gi|19911776	Granule-bound starch synthase	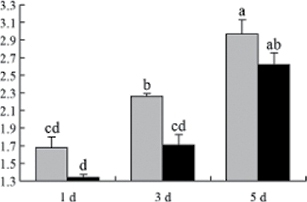
Energy metabolism								
OsUP5	355	40	13	35.9/27.4	5.41/5.39	gi|125528336	Dihydroxyacetone phosphate and d-glyceraldehyde-3-phosphate dehydrogenase	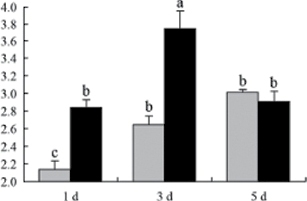
OsUP19	147	18	6	44.2/50.1	6.89/5.44	gi|115463933	Putative GDP dissociation inhibitor	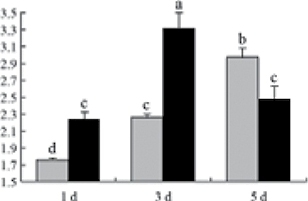
OsDP3	353	36	13	109.4/58.3	5.91/5.48	gi|115438749	ADP-glucose pyrophosphorylase large subunit	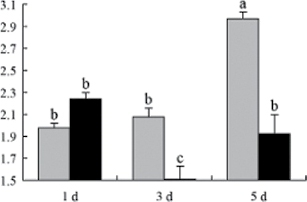
OsDP5	596	41	14	78.4/51.8	5.45/5.43	gi|115480571	UDP-glucose pyrophosphorylase	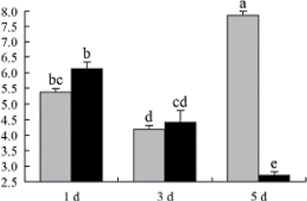
Oxidation								
OsUP3	490	41	10	34.2/27.3	5.43/5.42	gi|115452337	l-Ascorbate peroxidase	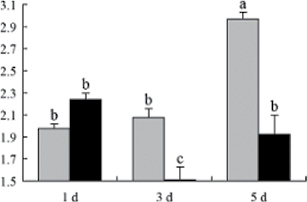
OsUP4	220	35	8	20.7/35.6	5.89/8.22	gi|115465579	Putative malate dehydrogenase	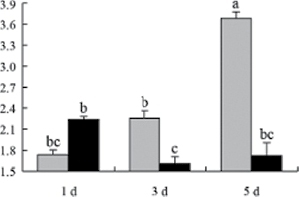
OsUP6	336	65	9	10.4/13.3	5.27/5.16	gi|115470941	Chain A, solution structure of thioredoxin type H from *Oryza sativa*	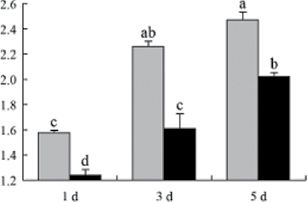
OsUP7	265	53	8	127.8/15.2	6.50/5.92	gi|115473931	Copper/zinc superoxide dismutase	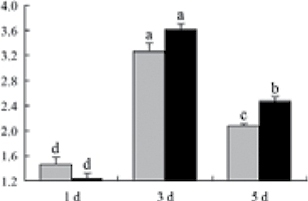
OsUP10	459	50	12	33.1/27.3	5.54/5.42	gi|115452337	Ascorbate peroxidase and cytochrome *c* peroxidase	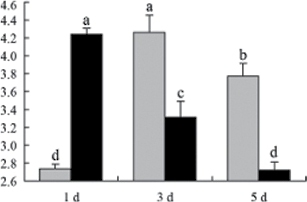
OsUP11	318	32	10	44.2/35.6	6.89/8.22	gi|115465579	Putative r40c1 protein, ricin-type beta-trefoil lectin domain-like protein	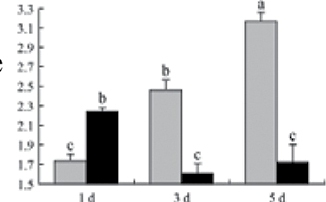
OsUP15	161	23	5	42.5/40.0	5.56/7.82	gi|115482392	Putative conserved protein	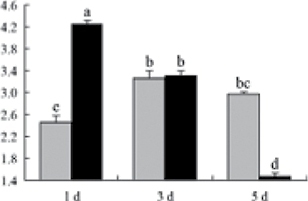
Heat shock								
OsUP1	276	29	9	18.2/18.1	5.51/4.57	gi|115463081	18.1kDa heat shock protein	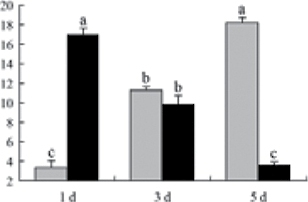
OsUP2	341	31	9	18.0/17.9	5.91/5.23	gi|18031726	17.9kDa heat shock protein	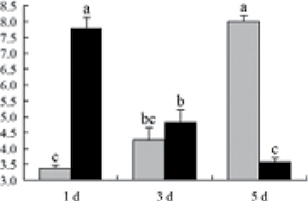
OsDP1	101	19	12	133.4/65.2	6.39/6.03	gi|115447567	Tetratricopeptide repeat domain protein (similar to heat shock protein STI).	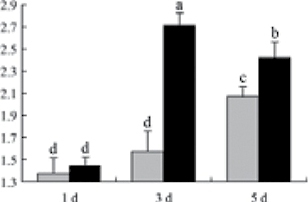
Transcript regulation								
OsUP9	167	27	5	42.5/28.5	5.40/5.45	gi|115464745	Basic/helix–loop–helix (bHLH) transcription factors	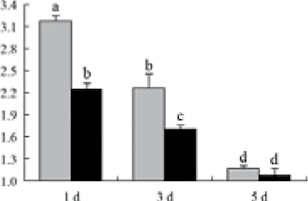

^*a*^ Spot experiment name corresponds to the spots in Figs 2 and 3. OsUP and OsDP indicate up- and down-regulated proteins, respectively.

^*b*^ Relative expression graphs of the protein spots after high temperature treatment in heat-sensitive and heat-tolerant rice lines. Mol. wt and pI indicate molecular weight and isoelectric point, respectively. Grey bars indicates heat-sensitive rice, and black bars indicates heat-tolerant rice. Spot volumes were analysed by Progenesis software. The fold change of up-regulated protein spot volumes was calculated by treatment/control, whereas the change fold of down-regulated protein spot volumes was calculated by control/treatment. From left to right, each bar indicates the fold change in protein spot volumes compared with control after exposure to high temperature for 1, 3, and 5 d. Values are presented as means ±SE. Error bars are from three spots in three independent gels.

**Fig. 4.**
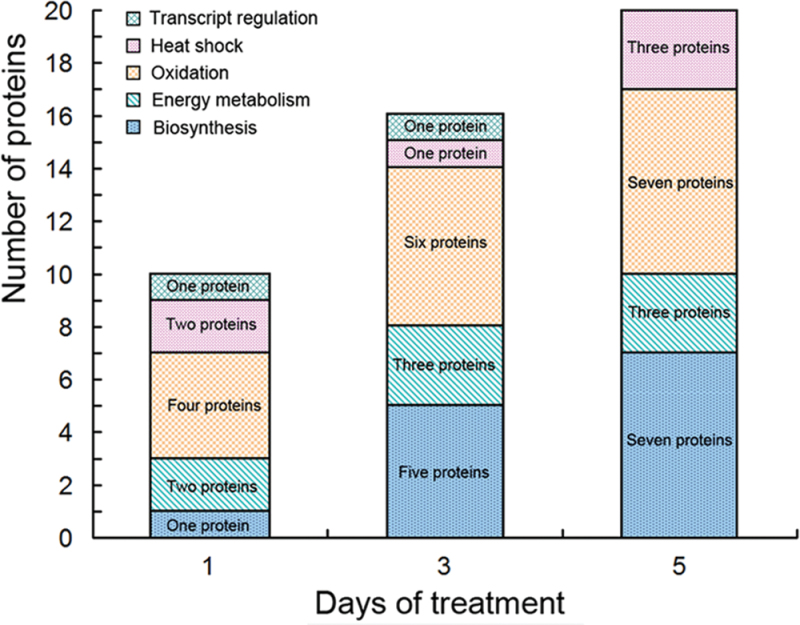
Protein numbers of all functional groups and the variations between high temperature treatments after 1, 3, and 5 d. The number of proteins that differed significantly between heat-tolerant and heat-sensitive rice lines were calculated for each functional group on each treatment day, and the numbers were calculated from each treatment time. (This figure is available in colour at *JXB* online.)

After 1, 3, and 5 d of high temperature stress, 10 proteins (OsDP6, OsUP5, OsUP19, OsUP6, OsUP10, OsUP11, OsUP15, OsUP1, OsUP2, and OsUP9), 16 proteins (OsUP13, OsUP18, OsDP2, OsDP4, OsDP6, OsDP8, OsUP5, OsUP19, OsDP3, OsUP3, OsUP4, OsUP6, OsUP10, OsUP11, and OsDP1), and 20 proteins (OsUP8, OsUP13, OsUP16, OsUP18, OsDP2, OsDP4, OsDP6, OsUP19, OsDP3, OsDP5 OsUP3, OsUP4, OsUP6, OsUP7, OsUP10, OsUP11, OsUP15, OsUP1, OsUP2, and OsDP1) were significantly up- and down-regulated in the heat-tolerant and heat-sensitive rice lines, respectively (Fig. 4 and Table 1). These results suggest that there is an increased number of differentially displayed proteins between heat-tolerant and heat-sensitive rice lines after high temperature treatments, and that these proteins are involved predominantly in oxidation and biosynthesis.

Of the 10 proteins involved in biosynthesis (Table 1), six (OsUP8, OsUP12, OsUP13, OsUP14, OsUP16, and OsUP18) were up-regulated in both heat-sensitive and heat-tolerant rice lines, and were involved in glutelin biosynthesis. The remaining four proteins were down-regulated in both rice lines; three of these (OsDP2, OsDP6, and OsDP8) were involved in starch biosynthesis, and one (OsDP4) in amino acid biosynthesis.

Of the four proteins involved in energy metabolism (Table 1), dihydroxyacetone phosphate (OsUP5) and a putative GDP dissociation inhibitor (OsUP19) were up-regulated in both heat-tolerant and heat-sensitive rice lines under high temperature stress, and they were up-regulated more significantly in heat-tolerant rice lines compared with heat-sensitive plants after 1 d and 3 d of high temperature stress. However, their expression was lower after 5 d of high temperature stress. In addition, ADP-glucose pyrophosphorylase large subunit (OsDP3) and UDP-glucose pyrophosphorylase (OsDP5) were both down-regulated under high temperature stress in both heat-tolerant and heat-sensitive rice lines. These proteins were down-regulated to a greater extent in heat-tolerant rice lines compared with heat-sensitive lines under high temperature stress for day 1, but a lesser extent on day 5.

All seven proteins involved in oxidation (Table 1) were up-regulated in both heat-tolerant and heat-sensitive rice lines. Of these, OsUP3, OsUP4, OsUP10, OsUP11, and OsUP15 were expressed at higher levels in heat-tolerant rice lines compared with heat-sensitive lines after exposure to high temperature stress for 1 d. In contrast, of the seven proteins, six (OsUP3, OsUP4, OsUP6, OsUP10, OsUP11, and OsUP15) were expressed at lower levels in heat-tolerant rice lines compared with heat-sensitive plants after exposure to high temperature stress for 5 d.

Interestingly, two heat shock proteins, 18.1kDa heat shock protein (OsUP1) and 17.9kDa heat shock protein (OsUP2), were up-regulated in both heat-tolerant and heat-sensitive rice lines (Table 1 and Fig. 3). However, prolonged stress caused these proteins to be down-regulated in both heat-tolerant and heat-sensitive rice lines, but expression was down-regulated more in the heat-tolerant lines. Another heat shock protein, tetratricopeptide repeat domain protein (OsDP1), was down-regulated in both heat-tolerant and heat-sensitive rice lines. However, they were more down-regulated in heat-tolerant rice lines compared with heat-sensitive plants during the entire high temperature stress treatment.

The protein involved in transcriptional regulation, the basic/helix–loop–helix (bHLH) transcription factor (OsUP9), was up-regulated in both heat-tolerant and heat-sensitive rice lines during high temperature stress.

A comparison of the expression profiles of the differentially expressed proteins involved in energy metabolism, oxidation, and heat shock between heat-tolerant and heat-sensitive rice lines showed that the expression of most proteins was triggered earlier in heat-tolerant rice lines compared with heat-sensitive plants. It is possible that the self-conserving metabolism was triggered earlier in heat-tolerant rice lines than in heat-sensitive lines after exposure to high temperature conditions.

### Real-time qRT–PCR analysis of genes encoding differentially expressed proteins

Real-time qRT–PCR was used to monitor the expression patterns of the genes encoding the differentially expressed proteins at the transcriptional level. Eight representative genes [namely pre-pro-glutelin (OsUP8), glutelin (OsUP12), starch debranching enzyme (OsDP6), l-ascorbate peroxidase (OsUP3), ADP-glucose pyrophosphorylase large subunit (OsDP3), thioredoxin type H (OsUP6), orthophosphate dikinase (OsDP4), and 18.1kDa heat shock protein (OsUP1)] were selected based on their possible functions and expression patterns in 2-DE analysis. The dbEST IDs in the NCBI database (www.ncbi.nlm.nih.gov/dbEST) and the PCR primers used to assess the eight representative genes are shown in Supplementary Table S1 at *JXB* online.

**Fig. 5.**
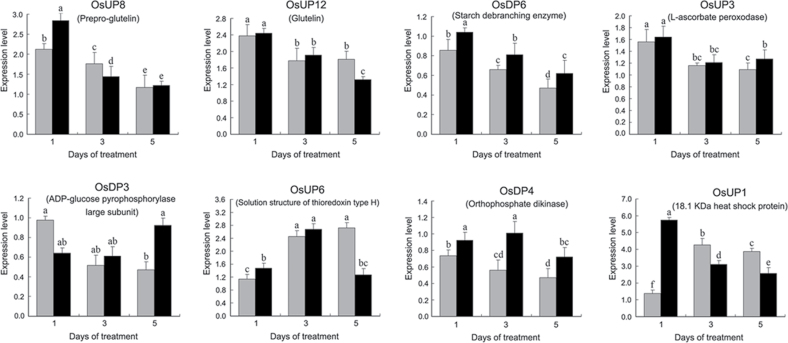
Expression of eight representative genes encoding the differentially expressed proteins as shown by real-time qRT–PCR analysis in heat-tolerant and heat-sensitive rice lines under high temperature for 1, 3, and 5 d. Rice caryopses were sampled from heat-tolerant and heat-sensitive rice lines after 1, 3, and 5 d of high temperature stress. Real-time qPCR was carried out by SYBR-green fluorescence using a 7500 Real-Time PCR Machine (Applied Biosystems). The threshold cycle (CT) values, representing the PCR cycle at which fluorescence passed the threshold, were generated using the ABI PRISM 7500 software tool. All data were normalized against the expression of the reference gene ACT1 (GenBank ID: AK100267). The relative expression levels were calculated using the comparative ΔΔCT method, and ΔΔCT= (C_T_, _Target_−C_T_, _Actin_)_time X_−(C_CK_, _Target_−C_CK_, _Actin_) _time X_. ‘Time X’ indicates 1, 3, and 5 d of high temperature stress. The mean expression values were calculated from triplicate measurements of each target gene.

A comparison of qRT–PCR analysis and 2-DE analyses suggested that the expression of genes encoding the differentially regulated proteins also changed in response to temperature stress. However, the changes in expression at each time point were different (Fig. 5 and Table 1). For example, the genes encoding pre-pro-glutelin (OsUP8), glutelin (OsUP12), and l-ascorbate peroxidase (OsUP3) showed the highest expression levels in both heat-tolerant and heat-sensitive rice lines after 1 d of high temperature stress; however, in 2-DE analysis, these genes showed the highest expression levels in both heat-tolerant and heat-sensitive rice lines after 3 d or 5 d of high temperature stress. These results suggest that the transcription of these genes was trigged earlier, resulting in a subsequent increase in protein expression. The different observation from real-time qRT–PCR and 2-DE analysis could be attributed to mRNA stability, splicing, translational regulation, post-translational processing, altered protein turnover, protein degradation, or a combination of these (Wei *et al.*, 2009).

### Genes encoding the differentially expressed proteins were mapped to the rice chromosomes

**Fig. 6.**
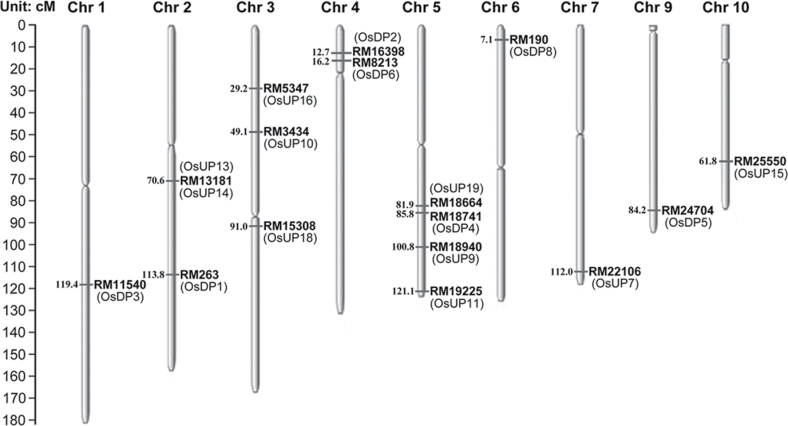
Rice chromosomal location of genes encoding differentially expressed proteins. The RILs and 887 SSR markers were used as the plant mapping population and mapping markers, respectively. If the PCR-amplified fragment of a target gene co-segregated with one or more markers in all the RILs, the target gene was considered to be in the same linkage group as the markers, and was mapped to the rice genome according to the locus of the markers.

Seventeen of the 25 genes co-segregated with 16 markers in the RILs and were mapped to different linkage groups on rice chromosomes 1, 2, 3, 4, 5, 6, 7, 9, and 10 (Fig. 6). The Michigan State University (MSU) locus numbers and the maker names are shown in Supplementary Table S2 at *JXB* online. Eight of the 25 genes could not be located in the rice genome because no co-segregating markers were found.

## Discussion

Proteomic analysis is an effective method to investigate the integral changes at the protein level in plants during environmental stress (Lee *et al.*, 2006; Yan *et al.*, 2006). Using proteomic methods, some proteins that respond to high temperature during rice grain development were identified, which will help with understanding the molecular mechanisms underlying the response of rice to high temperature during the ripening period. Rice embryos and endosperm undergo cell division and differentiation during the ﬁrst half of the ripening period, whereas the rice embryo reaches maturity and the endosperm undergoes matter accumulation and transformation during the second half of the ripening period (Hong *et al.*, 1995). Therefore, rice kernel formation differs in the ﬁrst and second half of the ripening period at both the physiological and molecular levels. To date, there have been no reports describing proteomic analysis during the first half of the ripening period or at the milky stage of the rice grain ripening phase. In this study, a comparative proteomic analysis between the heat-sensitive rice line XN0437S and the genetically similar heat-tolerant line XN0437T was performed to identify the functional proteins involved when rice is exposed to high temperature stress during the first half of the ripening period. These lines are progeny of the same parents and had very similar genomes, but show significant differences in grain weight after exposure to high temperature stress (Liao *et al.*, 2011). These lines were inbred specifically for this purpose. The use of these two rice lines in the present study ensures that the influence of non-target proteins and the noise from other parts of the plant genome were minimized, suggesting that the identified proteins correlated with the response to high temperature stress conditions.

### Influence of high temperature on glutelin-related proteins and starch-related enzymes, and differential patterns of heat-tolerant and heat-sensitive rice lines

Rice storage proteins are classified into glutelins, prolamins, globulins, and albumins, and are affected by high temperatures at the rice grain-filling stage in different cultivars (Lin *et al.*, 2005). Lin *et al.* (2010) reported that six glutelin-related proteins in rice caryopsis respond to high temperatures, and that their expression increases significantly under high temperature stress during the grain-filling stage. In this study, four glutelin-related proteins (OsUP8, OsUP12, OsUP13, and OsUP14) were identified and all four were found to be up-regulated in both heat-sensitive and heat-tolerant rice lines under high temperature stress during the first half of the ripening period. Moreover, two glutelin-related proteins (OsUP8 and OsUP13) in heat-sensitive rice lines showed significantly higher up-regulation compared with heat-tolerant lines under high temperature stress for 5 d. This suggested that high temperature conditions accelerated the maturity and ripening of rice grains, and that this effect was more apparent in heat-sensitive lines compared with heat-tolerant lines.

Starch quality is a crucial factor in determining the quality of rice grains. The content and fine structure of amylose and amylopectin in starch affect the physicochemical characteristics (such as viscosity) and texture properties of the rice grains (Muench *et al.*, 1997). During kernel development, granule-bound starch synthase (GBSS, Wx protein) and starch debranching enzymes are thought to play key roles in rice starch biosynthesis and show sensitivity to environmental conditions including high temperature (Kubo *et al.*, 1999; Jiang *et al.*, 2003; Lin *et al.*, 2005; Lin *et al.*, 2010). GBSS and starch debranching enzyme degrade in rice caryopsis under high temperature stress (Lee *et al.*, 2009; Lin *et al.*, 2010; Li *et al.*, 2011). In this study, two starch debranching enzymes (OsDP2 and OsDP6) and a GBSS (OsDP8) were measured and all three were down-regulated in both heat-sensitive and heat-tolerant rice lines under high temperature stress; however, the magnitude of the down-regulation in heat-sensitive rice lines was higher than that in heat-tolerant lines. This suggests that starch debranching enzymes and GBSS in rice caryopsis are some of the key proteins that maintain the normal development of heat-tolerant rice lines in high temperature environments.

### Energy metabolism-related proteins were down-regulated during assimilation and were up-regulated during dissimilation

GDP dissociation inhibitor (GDI) is a regulator that regulates the GTP/GDP-bound state and subcellular localization of G proteins. It also inhibits the GTPase activity of G protein itself and its effects on GDP dissociation (Pfeffer *et al.*, 1995; Yang *et al.*, 2006). GDI plays an important role in the response of rice to fungal pathogens, salicylic acid (SA), abscisic acid (ABA), and other stresses (Kim *et al.*, 1999; Yang *et al.*, 2006). In this study, a GDI (OsUP19) was found to respond to high temperature stress, and was up-regulated in both heat-sensitive and heat-tolerant rice lines under high temperature conditions. Interestingly, the levels of OsUP19 were highest in heat-tolerant rice at 1 d and 3 d, but were higher in heat-sensitive lines after 5 d of temperature stress. This suggests that the GDI in heat-tolerant rice was triggered and returned to normal earlier in heat-tolerant rice lines compared with heat-sensitive lines.

ADP-glucose pyrophosphorylase (ADPase) is an essential enzyme in starch granule synthesis that catalyses the conversion of sucrose to starch in the rice endosperm (Nakamura and Yuki, 1992). The speed of starch accumulation in the rice endosperm is related mainly to the activity of ADPase and its ability to cleave sucrose (Cheng *et al.*, 2005). In the presemt study, ADPase was down-regulated several folds in heat-sensitive rice, but only slightly in heat-tolerant rice under high temperature at 1 d and 5 d. Therefore, starch granule synthesis was probably less affected in heat-tolerant rice lines than in heat-sensitive lines.

UDP-glucose pyrophosphorylase (UDPase) is responsible for the synthesis and pyrophosphorolysis of UDP-glucose, and catalyses the conversion of glucose 1-phosphate and UTP into UDP-glucose, which then serves as a glycosyl donor for glutelin synthesis (Chen *et al.*, 2007). Lin *et al.* (2005) found that a UDPase was involved in the response of rice caryopsis to high temperature stress during caryopsis development, and was up-regulated between days 6 and 32 anthesis. In this study, a UDPase was identified that was down-regulated in both heat-tolerant and heat-sensitive rice lines under high temperature conditions. The protein expression continued to decrease in heat-tolerant rice lines, but exhibited a rapid increase in heat-sensitive lines after high temperature stress for 5 d. Therefore, the activity of UDPase in heat-tolerant rice lines could stimulate the recovery to normal conditions after a period of self-adjustment, whereas heat-sensitive rice lines did not demonstrate this capability for recovery.

d-Glyceraldehyde-3-phosphate dehydrogenase is a key enzyme in the glycolytic conversion of glucose to pyruvic acid that catalyses the oxidative phosphorylation of d-glyceraldehyde to 1,3-bisphosphoglycerate (Harris *et al.*, 1980). The enzyme activity of d-glyceraldehyde-3-phosphate dehydrogenase increases in photosynthetic tissues in response to light (Kelly and Gibbs, 1973). In this study, a protein homologous to d-glyceraldehyde-3-phosphate dehydrogenase was identified in rice caryopsis that was up-regulated in both heat-tolerant and heat-sensitive rice lines under high temperature. The up-regulation was higher in heat-tolerant rice lines compared with heat-sensitive lines after 1 d or 3 d of high temperatures.

### Proteins involved in oxidation were up-regulated differentially in two rice lines

Reactive oxygen species (ROS) can be either beneficial or harmful to cells and tissues. At physiologically low levels, ROS function as ‘redox messengers’ in intracellular signalling and regulation, whereas excess ROS induce the oxidative modification of cellular macromolecules, inhibit protein function, and promote cell death. Oxidative bursts induced by environmental stress trigger disturbances in the cellular redox balance, which are highly toxic. Plants have evolved complex regulatory mechanisms to prevent cellular injury by regulating the steady-state levels of ROS (Kelly and Gibbs, 1973; Circu and Aw, 2010). The peroxidase family plays a major role in regulating the levels of ROS, and serve as detoxifying enzymes and oxidize a wide variety of compounds in the presence of H_2_O_2_ (Teixeira *et al.*, 2004). Plant peroxidases perform functions such as the removal of H_2_O_2_ and the oxidation of toxic reductants (Faltin *et al.*, 2010). In this study, four proteins [an l-ascorbate peroxidase (OsUP3), a putative malate dehydrogenase (OsUP4), a copper/zinc superoxide dismutase (OsUP7), and a cytochrome *c* peroxidase (OsUP10)] were identified that could regulate ROS levels. The expression levels of these proteins were significantly higher in both heat-tolerant and heat-sensitive rice lines during the process of adaptation to high temperature stress. l-Ascorbate peroxidase, putative malate dehydrogenase, and cytochrome *c* peroxidase were all up-regulated to a significantly greater extent in heat-sensitive rice lines compared with heat-tolerant rice lines after 3 d of high temperature stress (Table 1). In contrast, the up-regulation scope of copper/zinc superoxide dismutase in the heat-sensitive rice line was significant lower than in the heat-tolerant line after 5 d of high temperature stress.

Thioredoxins (Trxs) are a multigene family of proteins that play a critical role in redox balance regulation via thiol–disulphide exchange reactions (Zhang *et al.*, 2011). In plants, Trxs have two major roles. First, Trxs can regulate the redox status of target proteins through thiol–disulphide exchange reactions. Secondly, they can enhance the heat shock resistance of plants via redox-independent mechanisms (Lee *et al.*, 2009; Park *et al.*, 2009). An apoplastic H-type thioredoxin is involved in salt stress and the regulation of the apoplastic ROS in rice (Zhang *et al.*, 2011). In the present study, thioredoxin type H (OsUP6) was identified and up-regulated in both heat-sensitive and heat-tolerant rice in response to high temperature. However, its expression was significantly higher in heat-sensitive rice lines compared with heat-tolerant lines throughout the high temperature stress period.

### Differential pattern of heat shock proteins in heat-sensitive and heat-tolerant rice lines

Heat shock proteins (Hsps) are molecular chaperones that regulate the folding and accumulation of proteins as well as their localization and degradation in all plant and animal species (Al-Whaibi, 2011). The significant up-regulation of the Hsps is a key part of the heat shock response, and is induced primarily by heat shock factors (HSFs) such as drought, salinity, cold and hot temperatures, and chemicals (Wu, 1995; Scarpeci *et al.*, 2008; Al-Whaibi, 2011). Small Hsps have been identified and are stimulated by high temperature stress. They are also up-regulated in rice caryopses during the grain milky stage (Lin *et al.*, 2005). Two Hsps (OsUP1 and OsUP2) were monitored, and it was found that both were up-regulated in heat-sensitive and heat-tolerant rice caryopses. Furthermore, the expression of OsUP1 and OsUP2 in heat-tolerant rice lines was triggered rapidly. The maximal expression was observed after 1 d of high temperature stress, and their expression decreased with prolonged stress. In contrast, the expression of OsUP1 and OsUP2 in heat-sensitive rice was triggered later and increased gradually, reaching a maximum on the fifth day of high temperature stress.

### Possible processes that regulated the expression of differentially expressed proteins during high temperature stress in rice at the early milky stage

**Fig. 7.**
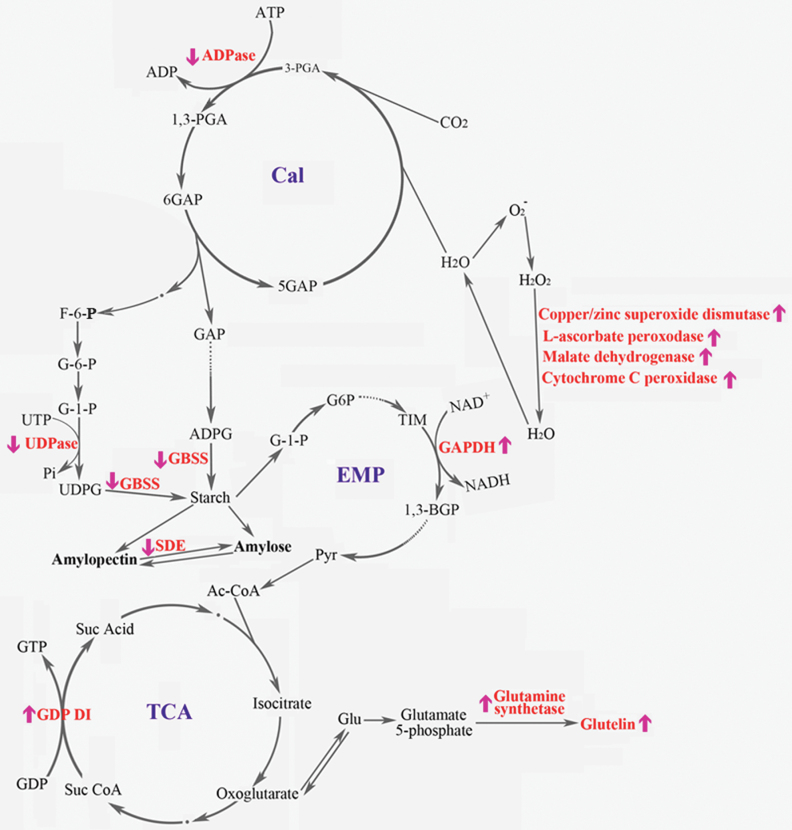
Putative functions of the differentially expressed proteins in metabolic processes of rice grain development at high temperatures. Proteins of known function are shown in red text. Up-regulated proteins are marked with ↑, and the down-regulated proteins are marked with ↓. Cal, Calvin cycle; EMP, glycolytic pathway; TCA, tricarboxylic acid cycle; ADPase, ADP-glucose pyrophosphorylase (OsDP3); UDPase, UDP-glucose pyrophosphorylase (OsDP5); GBSS, granule-bound starch synthase (OsDP8); SDE, starch debranching enzyme (OsDP6); GDI, putative GDP dissociation inhibitor (OsUP19); GAPDH, d-glyceraldehyde-3-phosphate dehydrogenase (OsUP5); GAP, glyceraldehyde-3-phosphate; G-6-P (G6P), glucose 6 phosphate; UDPG, uridine diphosphoglucose; Pyr, pyruvate; Ac-CoA, acetyl-CoA; Suc Acid, succinic acid; Suc CoA, succinic-CoA; TIM, triose phosphate enzyme; 1,3-BGP, 1,3-diphosphoglyceric acid; Glu, glutamate. (This figure is available in colour at *JXB* online.)

Based on the functions of the differentially expressed proteins and the expression patterns suggested by comparative proteomics and real-time PCR, a possible mechanism is proposed by which these proteins function in response to heat shock in the plant cell (Fig. 7). Initially, heat shock disrupts the redox balance (Yang *et al.*, 2004) and the related peroxidase, l-ascorbate peroxidase (OsUP3), malate dehydrogenase (OsUP4), copper/zinc superoxide dismutase (OsUP7), and cytochrome *c* peroxidase (OsUP10) are all up-regulated. The signal of this redox imbalance is transduced to the Calvin cycle (Cal cycle), and ADP-glucose pyrophosphorylase (OsDP3, which plays a role in the Cal cycle) is down-regulated. This affects the energy-related product glyceraldehyde-3-phosphate (GAP), which limits the material available for starch synthesis. This causes the UDPase (OsDP5) and GBSS (OsDP8) to regulate the catalysis of glucose-1-phosphate (G-1-P) to starch in the cytoplasm and chloroplasts. The interchange between amylopectin and amylase is affected by the quantity of synthetic starch, and starch debranching enzyme is down-regulated. In response to high temperature stress, the glycolytic pathway (EMP) accelerates, as does the key enzyme d-glyceraldehyde-3-phosphate dehydrogenase (OsUP5) that catalyses conversion of triose phosphate to 1,3-diphosphoglyceric acid (1, 3-BGP) in the EMP. This suggests that starch in plant cells is synthesized less and consumed more under high temperature conditions compared with normal.

The faster EMP under high temperature conditions results in increased production of pyruvate (Pyr). As a substrate signal of the tricarboxylic acid (TCA) cycle, high levels of Pyr accelerate the TCA cycle process, which increases the conversion of succinic-CoA (Su CoA) to succinic Acid (Su Acid) that is catalysed by the GDP (OsUP19). This suggests that GDI is up-regulated. Oxoglutarate is a substrate of glutelin synthesis in the TCA cycle, and is up-regulated when the TCA activity is increased. The up-regulation of oxoglutarate suggests that glutelin synthesis and glutamine synthetase (OsUP16) are up-regulated. The differentially expressed proteins regulate the above processes, supporting the present observation that the accumulation of starch in rice grains under high temperature stress was less than that under normal temperature conditions, while the glutelin accumulation of rice grains was accelerated under high temperature stress.

### Genes encoding the differentially displayed proteins were mapped to rice chromosomes

To date, only a small number of genes involved in the high temperature stress response of rice at the milky stage have been mapped to rice chromosomes due to the difficulty of selecting phenotypic traits of rice that are related to high temperature. A previous study identified the genetic locus of rice that is related to high temperature stress at the milky stage (Liao *et al.*, 2012). In this study, 17 genes involved in the response of rice to high temperature stress at the milky stage were mapped to different linkage groups on the rice chromosomes by identifying co-segregating conditions between SSR markers and PCR-amplified fragments from the identified genes. However, eight of the 25 identified genes could not be located on the rice genome because no co-segregating markers were found. The function, molecular weight, and pI of the proteins according to the LOC_ID (Supplementary Table S2 at *JXB* online) were further compared with the proteomic results from 2-DE. The characteristics of 12 proteins (OsUP7, OsUP10, OsUP13, OsUP14, OsUP16, OsUP18, OsUP19, OsDP2, OsDP3, OsDP4, OsDP6, and OsDP8) coincided with the results from 2-DE, but those of the other five proteins did not. Therefore, it is possible that these five proteins were in the same linkage groups as the co-segregating markers, but not at their exact locus. The genes mapped to rice chromosomes could provide useful information for further studies on fine genetic mapping and cloning the full-length genes of rice that regulate the heat tolerance trait.

### Genes responding to high temperature stress were differentially regulated at the transcriptome and proteome level

Comparative analysis of the results of this study and previous transcriptional analysis data (Liao *et al.*, 2012) showed that the number of genes responding to high temperature in the proteome was different in the transcriptome. Furthermore, their function, classification, and regulation were also different. Three genes from the transcriptome (OsRH65, OsRH56, and OsRH25) and three from the proteome (OsDP1, OsDP6, and OsDP7) were present in the same region on rice chromosomes 2, 4, and 7, respectively. Nevertheless, the genes in rice that respond to high temperature stress are regulated differentially at the transcriptional and proteomic levels, suggesting that the approaches of the transcriptome and proteome are highly complementary, consistent with the findings of a previous study (Møller *et al.*, 2011; Yun *et al.*, 2012).

## Supplementary data

Supplementary data are available at *JXB* online.

Figure S1. The rice chalkiness area increased in both heat-tolerant and heat-sensitive rice lines exposed to high temperature stress for 1, 3, and 5 d.

Figure S2. Histogram of variable grain weight and net photosynthetic rate of rice under high temperature in both rice lines.

Table S1. Specific primers and dbEST IDs of eight representative genes encoding the differentially displayed proteins used in relative quantitative real-time reverse transcription–PCR.

Table S2. The Michigan State University (MSU) locus numbers and PCR primer sequences of co-segregating markers of 17 mapped genes in rice that respond to high temperature stress at the early milky stage.

Supplementary Data
