# Supplementary material for: Comparative proteomic analysis of differentially expressed proteins in the early milky stage of rice grains during high temperature stress
Source: J Exp Bot. 2013 Dec 27;65(2):655–71. doi: 10.1093/jxb/ert435 (PMC3904723; doi:10.1093/jxb/ert435)
Supplement: Supplementary Data [file supp_65_2_655__index.html]

Comparative proteomic analysis of differentially expressed proteins in the early milky stage of rice grains during high temperature stress — Comparative proteomic analysis of differentially expressed proteins in the early milky stage of rice grains during high temperature stress — Supplementary Data 

# Comparative proteomic analysis of differentially expressed proteins in the early milky stage of rice grains during high temperature stress

## Supplementary Data

Data files

**Files in this Data Supplement:**

- Supplementary Data - Supplementary Data
