# Supplementary material for: Comparative proteomic analysis of differentially expressed proteins in the early milky stage of rice grains during high temperature stress
Source: J Exp Bot. 2013 Dec 27;65(2):655–71. doi: 10.1093/jxb/ert435 (PMC3904723; doi:10.1093/jxb/ert435)

Comparative proteomic analysis of differentially expressed proteins in the early milky stage of rice grains during high temperature stress

Jiang-Lin Liao [1, 2], Hui-Wen Zhou [2], Hong-Yu Zhang [2], Ping-An Zhong [2], Ying-Jin Huang [1, 2] *

[1] Key Laboratory of Crop Physiology, Ecology and Genetic Breeding (Jiangxi Agricultural University), Ministry of Education, Jiangxi Province 330045, China

[2] Key Laboratory of Agriculture responding to Climate Change (Jiangxi Agricultural University), Nanchang City, Jiangxi Province 330045, China

Supplementary Tables and Figures

Supplementary Table S1

Specific primers and dbEST IDs of eight representative genes encoding the differentially displayed proteins used in relative quantitative real-time reverse-transcription PCR.

Protein Exp. name	dbEST ID	Forward primer (5'->3')	Reverse primer (5'->3')	Product size (bp)
OsUP1	66844078	AAAGGAGCCGGGAGCAAGAG	TCTCACAACCAGTTTGCACGC	303
OsUP3	15596612	AGGGTACGTGTAGGCCTCGT	GTTCGTCGCAGTTGGTGTGG	241
OsUP6	15597371	TGCTGAGGCTGACAAGGTCG	ACACCCAAGCCAAACTGACG	195
OsUP8	1472831	GGGGAGTGAATGCACCGAAC	AACGGAGAGCTTCGTCGTGG	256
OsUP12	16976838	ATGGGAGTGGCTGCCCTAAC	TGTCCACGACGAAGCTCTCC	317
OsDP3	1474432	TGGACCCCCTGAGTGTTTGC	CGAGCAGCCTCCAAAGTTCG	375
OsDP4	1472321	GGCACGAGGCAGCTTTAGTG	TTGCGGCCTCTCTCTGTAGC	226
OsDP6	15596862	AGTCAGCATACGAGGCGTCC	AAAGCATGCCGAAACCTGGG	287

Supplementary Table S2

The Michigan State University (MSU) locus numbers and PCR-primer sequences of co-segregating markers of 17 mapped genes in rice that respond to high temperature stress at the early milky stage.

Exp. name	MSU locus numbers	Marker name	Forward primer sequence (5′-3′)	Reverse primer sequence (5′-3′)	Amplication size (bp)
OsUP13	LOC_Os02g0453600	RM13181	TAGGAAGGCAAGCAATGAACAGG	CGAGTGTATGGTCAGGTTGATGG	263
OsUP14	LOC_ Os02g0453600	RM13181	TAGGAAGGCAAGCAATGAACAGG	CGAGTGTATGGTCAGGTTGATGG	263
OsUP16	LOC_Os03g12290	RM5347	GTGCGCCGGAATTACGCTTCC	GCAGCACGCAGTTCTCCTTCTCC	167
OsUP18	LOC_Os03g0427300	RM15308	ATCCACCGTCGTTTCTTCTTTCC	AGTGCTGCCCAATTAATGTACGG	246
OsDP2	LOC_Os04g0164900	RM16398	GCATGTAACTTGAGAGTCATGG	ACGATCTTTAGTCCAGGTTGG	337
OsDP6	LOC_Os04g0164900	RM8213	TGTTGGGTGGGTAAAGTAGATGC	CCCAGTGATACAAAGATGAGTTGG	179
OsDP8	LOC_Os06g04200	RM190	GCTACAAATAGCCACCCACACC	CAACACAAGCAGAGAAGTGAAGC	144

OsUP19	LOC_Os05g34540	RM18664	GAACTTATTTAGGGTCGGATGG	CGTCGACGTCAATATGATGG	388
OsDP3	LOC_Os01g0633100	RM11540	GAAGCTCATGGGCCACATCAAGG	GCGCTTCCGCTCTCTAAGGTTCC	196
OsDP5	LOC_ Os09g0553200	RM24704	CACCTCCACCTCTCGCATTCC	CTCCGTGTGCTTCTTGCAGTCC	172
OsUP7	LOC_Os07g46990	RM22106	CAAAGGACCCTCCCATAACACC	ATAAGGTCTCTCGCCTTGGATGG	122
OsUP10	LOC_Os03g0285700	RM3434	AGAGAAATGCCAGCTTTGACTGC	CCAGCTAGGATGTTGAAGGATCG	144
OsUP11	LOC_ Os05g0574400	RM19225	ATAACCCTCTCTTTCCCTTTGG	GAGAGGAGGGAGGAGGAAGC	188
OsUP15	LOC_Os10g32680	RM25550	AAGGAGGAGTACTTAAGGGTAGTGG	TTTATAGGAGCTAAGGTGGAGTGG	346
OsDP4	LOC_Os05g0405000	RM18741	AATGAGGAGGAGGAGGAGAAGC	ACTTACTGCATTACAGTCGTGTGG	321
OsDP1	LOC_Os02g43020	RM263	AATCTATGGACCTGGGAGGAACC	TGACGAGAGTGCTACGTTTGAGC	235
OsUP9	LOC_Os05g42230	RM18940	GATCGATCAGTCAACCAAGAAGC	AGAAGAGGTATCCAAAGGCAAGG	235

Supplementary Figure S1

The rice chalkiness area increased in both heat-tolerant and -sensitive rice lines exposed to high temperature stress for 1, 3 and 5 days. After maturity, rice seeds from the same region (middle to bottom part) of labelled ears from controls and treatment groups were harvested. Samples were sun-dried and grain chaffs were removed artificially.

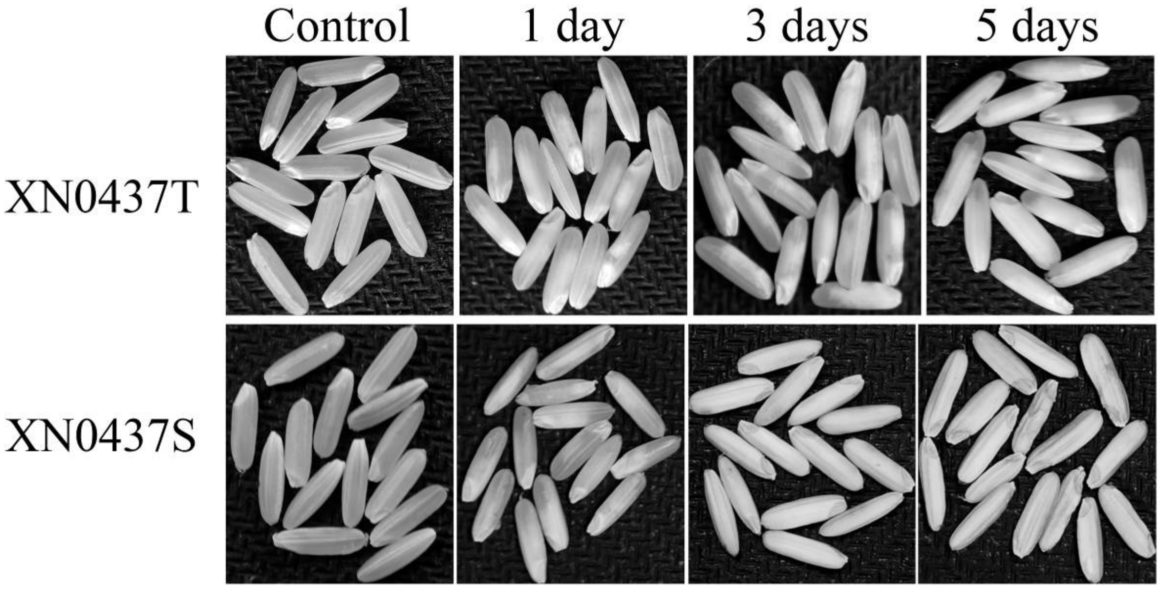

Supplementary Figure S2

Effects of high temperatures on grain weight (A) and net photosynthetic rate (B). After maturity, grains were harvested from ten labelled rice ears. They were then sun-dried, and unfertilized spikelets were removed. They were then weighed, and the percentage of variation of grain weight (VGW) between treatment (GWt) and control (GWc) was calculated using the formula VGW (%) = (GWt/GWc) × 100%. The net photosynthetic rate (NPR) of rice flag leaves was assessed on the 2nd day after treatment. Three flag leaves were measured for each sample

and the average values were calculated. The percentage variation in NPR between control (NPRc) and treated plants (NPRt) was calculated by the formula VNPR (%) = (NPRt/NPRc) × 100%. Three biological replicates were performed per sample. Analyses of significant differences in VGW and VNPR between heat-tolerant and -sensitive rice lines were carried out. Different lowercase letters on the top of bars indicate a significant difference at the 5% level and the same lowercase letters on the top of bars indicate no difference between heat-tolerant and -sensitive rice lines.

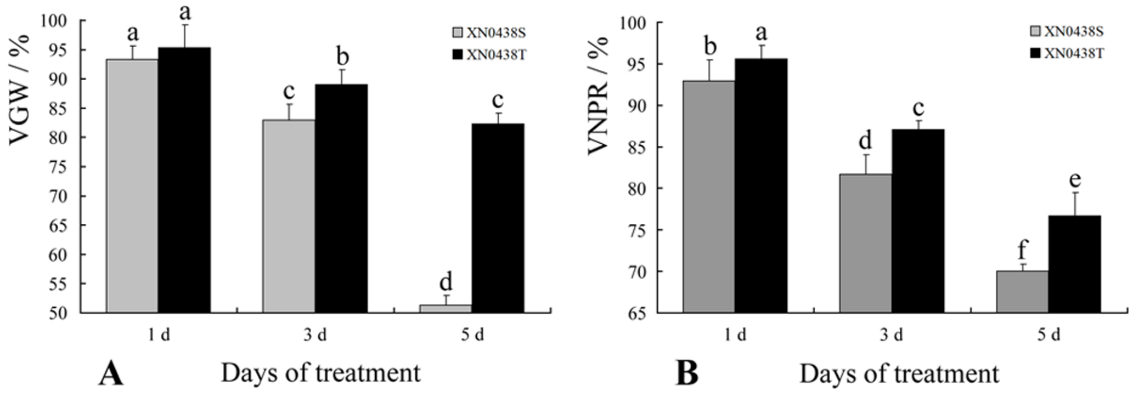

Supplement: Supplementary Data [file supp_ert435_jexbot101444_file001.pdf]
